# Prevalence and Relationships among Physical Activity Policy, Environment, and Practices in Licensed Childcare Centers from a Manager and Staff Perspective

**DOI:** 10.3390/ijerph17031064

**Published:** 2020-02-07

**Authors:** Jennifer McConnell-Nzunga, Louise C. Mâsse, E. Jean Buckler, Valerie Carson, Guy E. Faulkner, Erica Y. Lau, Heather A. McKay, Viviene A. Temple, Luke Wolfenden, Patti-Jean Naylor

**Affiliations:** 1 Child Health BC, 4088 Cambie St #305, Vancouver, BC V5Z 2X8, Canada; jsmcconn@uvic.ca; 2 School of Exercise Science, Physical and Health Education, University of Victoria, 3800 Finnerty Road, Victoria, BC, V8P 5C2, Canada; vtemple@uvic.ca; 3 School of Population and Public Health, University of British Columbia, 2329 West Mall, Vancouver, BC V6T 1Z4, Canada; lmasse@bcchr.ubc.ca (L.C.M.); jean.buckler@bcchr.ca (E.J.B.); 4 British Columbia Children’s Hospital Research Institute, 4480 Oak St., Vancouver, BC V6H 3N1, Canada; 5 Faculty of Kinesiology, Sport, and Recreation, 3-100 University Hall, Van Vliet Complex, University of Alberta, Edmonton, AB T6G 2H9, Canada; vlcarson@ualberta.ca; 6 School of Kinesiology, University of British Columbia, 6081 University Boulevard, Vancouver, BC V6T 1Z1, Canada; guy.faulkner@ubc.ca; 7 Department of Family Practice, Faculty of Medicine, University of British Columbia, 5950 University Boulevard, Vancouver, BC, V6T 1Z3, Canada; erica.lau@ubc.ca (E.Y.L.); heather.mckay@ubc.ca (H.A.M.); 8 School of Medicine and Public Health, University of Newcastle, University Drive, Callaghan, NSW 2308, Australia; Luke.Wolfenden@hnehealth.nsw.gov.au

**Keywords:** physical activity, fundamental movement skills, physical literacy, childcare, early childhood educators, policy, practice, licensing

## Abstract

Physical activity (PA) is critical to early childhood health and development, and childcare is a key setting for establishing physically active play. In British Columbia (BC), a provincial standard for active play in childcare was enacted, identified here as the Active Play (AP) standard. Pragmatic constraints limit real-world data collection for evaluating policy impact. We explored whether information about policies, practices, and the environment varied when it was collected from managers or staff. Surveys were distributed to BC childcare centers before AP standard enactment to ascertain current PA and fundamental movement skill policies and practices. The full sample (*n* = 1037 from 625 facilities) and a subsample of paired managers and staff (*n* = 261 centers) were used to explore agreement across managers and staff in reported prevalence and relationships among indicators. The policy prevalence and relationships for active play and outdoor play variables were relatively similar for manager and staff data, although the matched data had modest agreement and less than optimal intraclass correlations. The prevalence of manager-reported PA policies ranged from 47% for screen-time limits to 77% for fundamental movement skill activities. The manager and staff data highlighted indoor and outdoor space as a primary factor in AP standard adherence. With reliance on sampling staff unfeasible, it appears that the manager data may adequately describe the policies and practices of childcare providers with some notable issues.

## 1. Introduction

Physical activity (PA) is critical to early childhood development. Engaging in PA in the early years is associated with a number of physical, psychological, and social health benefits, as well as improved cognitive and language development [1]. In addition, participating in sufficient PA during the early years influences PA later in life [2], as it promotes the acquisition of fundamental movement skills (FMS) during this important developmental stage [3]. FMS are foundational movement skills that can be further developed into activity- or sport-specific skills and are subdivided into locomotor (e.g., running, hopping), manipulative (e.g., throwing, catching), and balance skills [4]. Importantly, strong FMS in early childhood is associated with greater PA in adolescence [5] and adulthood [6]. This increase in PA participation is thought to be driven by a hypothetical proficiency barrier, where a minimum skill capability is needed to engage in a wide variety of activities, and physical and psychological features may mediate an individual’s foundational skill capacity [7,8]. National surveys from 2009 to 2015 indicate that 61.8% and 24.4% of three- and four-year-olds meet the recommended 180 min of PA and spend less than 1 h on screen time per day, respectively [9]. In contrast, movement skill proficiency is lower than anticipated in Canada (mean locomotor and manipulative skills below the 25th percentile [10]) and internationally [11,12]) based on normative data collected 20–25 years ago, indicating a possible population-wide decline in skill proficiency [13,14,15].

Childcare centers are an important setting to enhance PA opportunities for young children, as more parents are accessing childcare facilities than ever before, especially in developed countries [16]. In Canada, the number of regulated childcare spaces has tripled from 1992 to 2014 [17]. More than half (54%) of Canadian parents with children under five years of age report using childcare, and of those children, 70% are in full time (at least 30 h a week) childcare [18]. This leaves less time outside of childcare for PA participation. Unfortunately, research indicates that the PA during childcare is insufficient (i.e., preschoolers are getting between 12 and 14 min of moderate-to-vigorous PA per day) and not supportive of the development of FMS [19,20,21]. The childcare center accounts for 37% of the variance in children’s vigorous PA [22], highlighting the need to implement policies for improving the amount and quality of PA that is provided.

In the United States, state-level childcare policy has been associated with improvements in childcare practices aimed at increasing children’s PA behavior as well as modifications in fixed play environments and improved training and education of childcare providers [23]. Assessing compliance with state-level regulations is important, as it has been associated with improved PA [24]. In contrast, a recent systematic review found no associations between the presence of facility-level PA policies and activity time, as measured by either service quality by the Environment and Policy Assessment and Observation (EPAO) tool or existence of a PA policy; however, in half of the studies, quality of programming was associated with PA [25]. While there is some evidence to suggest that similar factors may influence the implementation of childcare policies at the state- and facility-levels (e.g., provision of in-person training) [26], the context into which facility-level policies are implemented likely matters. Specifically, there is a need to account for whether facility-level policies are implemented in a state-regulated context and whether the facility has the resources and environment (e.g., indoor or outdoor space) to implement the PA policy.

Evaluating the impact of state-level policies on facility-level policies and practices in childcare settings is important from both a research and public health policy surveillance perspective. Current measurement tools, such as the EPAO [27] and the Environment and Policy Assessment and Observation Self-Report (EPAO-SR) [28], have primarily been used to assess childcare facility-level policies and practices in research studies where researchers have the resources to either conduct observations or administer questionnaires to both childcare managers and staff. However, in the real-world pragmatic context of the current study, our research team encountered a number of limitations in using the EPAO-SR protocols to explore how a provincial policy (Director of Licensing Standard of Practice for Active Play—AP standard, see Appendix A, Table A1) impacted the policies and practices of childcare providers. The most challenging component of the EPAO-SR is that it requires a multi-level survey—meaning that managers (level 1) and two staff members (level 2) need to complete the survey (with one staff member completing it twice). While a sampling frame could be designed from publicly available lists for the recruitment of managers (level 1 sampling frame) there was no publicly available list for recruiting staff (level 2 sampling frame). As a result, we had to rely on managers to distribute the survey to staff, and consequently, staff recruitment was lower. Additionally, staff turnover in childcare facilities is high, making it difficult to match data collected over different waves of data collection [29]. A specific interest of this study was to determine whether our understanding of how policies and environmental resources are associated with practice changes and whether information about practices in a given childcare setting is different when it is collected from the manager or staff as postulated in previous publications comparing policy to practice [30,31]. There is a need to establish a more pragmatic surveillance approach for monitoring the impact of state-level policies on childcare policies and practices.

The Good Start Matters study is a five-year prospective mixed-methods study examining the effects of implementing a provincial standard and capacity building in British Columbia, Canada, on PA in licensed childcare settings. We used the baseline data from the Good Start Matters study to explore whether information about policies, practices, and the environment varied when it was collected from managers or staff. Specifically the primary objectives of this paper were the following: (1) to determine whether descriptions of childcare environments and prevalence estimates of practices related to PA, FMS, and sedentary time were significantly associated with the data provider (managers or staff) and (2) to compare whether the associations between policies and PA environment and PA, FMS, and sedentary behavior practices were similar when practices and descriptors of policies and PA environments were collected from managers versus staff.

## 2. Materials and Methods 

### 2.1. Study Design

Baseline data from the Good Start Matters study were analyzed for this descriptive cross-sectional study using a survey developed to examine policy and practices in licensed childcare settings. The Good Start Matters study, taking place over 5 years, aims to track the implementation and impact of the Director of Licensing Standards of Practice for Active Play (AP standard; see Appendix A), which were enacted by the British Columbia provincial government in 2017. These standards aim to increase active play in regulated childcare facilities through outdoor play time, opportunities for children to participate in activities to develop physical literacy (defined as the motivation, confidence, physical competence, knowledge, and understanding to value and take responsibility for engagement in PA for life [32]) and FMS, reduced sitting time, modeling, and the implementation of facility AP and screen-time policies. Facilities are audited approximately every 18 months. Licensing officers log any contraventions and identify goals and a timeframe within which the childcare provider is expected to bring their facility into compliance [33]. The implementation of the AP standard presented the opportunity for a natural experiment and our research team was funded to examine the impact of the AP standard over time. During baseline, prior to enactment of the AP standard, we used a multi-level recruitment and data collection strategy based largely on EPAO-SR methods [28] to collect information on childcare policies, practices, and environments.

### 2.2. Participants

Eligible participants included managers and staff of licensed childcare centers serving children 3–5 years of age across British Columbia (BC), Canada. Recruitment took place from August 2015 to September 2016, prior to the implementation of the AP standard in 2017. Recruitment strategies included initial emails from licensing officers and direct mail, email, and phone calls from the research team using publicly available facility contact information as well as invitations distributed through childcare resource and referral agencies and early childhood educator newsletters. Childcare center managers and staff that did not respond to initial and follow-up email invitations were sent a paper copy of the survey with a pre-paid postage-stamped envelope for return. Managers who had not yet responded also received phone calls from our research team and were offered the choice of electronic or paper survey for themselves and their staff. The study was approved by the University of Victoria and University of British Columbia Harmonized Research Ethics Review Board (BC16-128 and H18-01434).

In about 4% of the facilities, more than one manager responded to the survey. In total, 25 manager responses were duplicate or triplicate from the same facility. As a result, thirteen cases were dropped in order to match one manager response to one facility. All duplicate and triplicate cases were processed as follows: if responses were identical, then one of the manager surveys for the facility was randomly selected, and the other(s) were dropped; if responses were missing on one manager survey, then the manager survey with missing responses was dropped; and if the responses showed disagreement, then all of the manager surveys for that facility were dropped.

### 2.3. Measures

Manager and staff completed a self-report survey based on questions adapted from the validated EPAO-SR childcare nutrition and PA instrument [28], including questions about daily practices, the new AP standard, and BC-specific childcare facility demographic characteristics. The EPAO-SR questions were adapted to ensure that the questions measured the policies and practices targeted by the AP standard. Managers and staff self-reported practices related to PA, FMS, and sedentary activities in their childcare facilities, and managers reported on the policies and environments related to PA, FMS, and sedentary activities. The AP standard addresses physical literacy explicitly, and while the survey does include questions that relate to physical literacy (e.g., FMS, PA opportunities), it does not specifically address opportunities to develop all components of physical literacy (e.g., motivation).

#### 2.3.1. Demographics

Respondents were asked about their age, gender, education, years of service, and role. In addition, details about childcare size (number and ages of children served), type, and staff/child ratio were collected.

#### 2.3.2. Daily Practices

Managers and staff indicated on a 5-point Likert-scale from “Daily” to “Rarely/Never” how often children participated in the following practices in their childcare program: engaged in at least 120 min of active play and PA per day (60 min for 1/2 day), spent 30 min or less on screens per day, took part in daily activities that develop FMS (a component of physical literacy), did not sit for prolonged periods (e.g., in a stroller, high chair, board games, crafts, etc.), and engaged in at least 60 min of outdoor active play per day. Responses were dichotomized into “Daily” and “Less than daily” for analysis.

#### 2.3.3. Policies

Managers were asked whether their facility had a policy that includes a statement about the following: the amount of active play time for children, the amount of staff-led active play time, the amount of time children spend outdoors each day, the amount of time children can play with screens (watch television/video each day, computer, games), the amount of facilitated activities targeting locomotor skills (e.g., running, hopping, jumping) offered, the amount of activities targeting balance offered, the amount of activities targeting manipulative skills (throwing, catching, kicking, etc., which in our survey were described as coordination skills based on sector feedback) offered, breaking up prolonged sitting time with activity, and the amount of unfacilitated play/free play. Managers selected from “No”, “Yes, not written policy, but general practice”, “Yes, written policy”, or “N/A”. Responses were dichotomized in to “Yes” (“Yes, written policy”) and “No” (“Yes, not written policy, but general practice”, “No”, or N/A”). A facility was considered to have an FMS Policy if they had a written policy for any one of the following: the amount of staff-led active play time, the amount of facilitated activities targeting locomotor skills (e.g., running, hopping, jumping) offered, the amount of activities targeting balance offered, and the amount of activities targeting manipulative skills (throwing, catching, kicking, etc.) offered.

#### 2.3.4. Environment

Managers and staff rated their outdoor space on a 4-point Likert-scale from “Space for large group running games (e.g., tag with entire group)”, “Space for small group (2–3 children) running games”, and “Only space for individual running/skipping/hopping” to “No space for running games (individual or group)” and their indoor space on a 5-point Likert-scale from “Room for all physical skills including running”, midpoint category, “Room for limited movement activities, e.g., walking, skipping, hopping, jumping, etc.”, midpoint category, to “No room, only able to use for quiet play”. These items were dichotomized into “Space for large group running games indoors” or not and “Space for large group running games outdoors” or not.

### 2.4. Statistical Analysis

Chi-square statistics were computed to compare whether manager and staff responses about practices and description of the environment differed. Intraclass correlations (ICC) between matched manager and staff responses were calculated to assess consistency. There are no standard values for acceptable reliability using ICC, but commonly, ICC values of less than 0.5 are considered poor reliability, values between 0.5 and 0.75 are considered moderate reliability, values between 0.75 and 0.9 are considered good reliability, and values greater than 0.90 are considered excellent reliability [34]. We calculated one-way random effects ICC for absolute agreement of PA practices as $\frac{MS_{R}\;-\;MS_{W}}{MS_{R}+\left(k+1\right)MS_{W}}$ where MSR = mean square for rows, MSW = mean square for residual sources of variance, and k = number of raters [29,30]. We assessed the ICC of PA practice variables as both dichotomized and in their original 5-point scale.

Associations between practices with policy and aspects of the environment were analyzed with generalized logistic models for manager data and with generalized logistic mixed-effects models (GLMER) for staff. GLMER was used for the staff data to account for the nested structure of the data, meaning that some facilities had multiple staff responses per facility. As all the practices were dichotomized, the analyses used the binomial family of models. Level of significance was set at *p* < 0.05. For parsimony, non-significant effects were removed from the final model. All statistical analyses and data manipulation were conducted using RStudio version 1.1.447 (RStudio, Boston, MA, USA) [35]. As 55% of the responses received from managers did not have a corresponding staff response, the manager data was analyzed with the full sample, and a subsample of those manager responses were matched to staff by facility. Fewer staff responses were lost when matched by facility (20%), so the staff sample was analyzed only once and included only those matched with a manager response from the same facility.

## 3. Results

**Figure 1:**
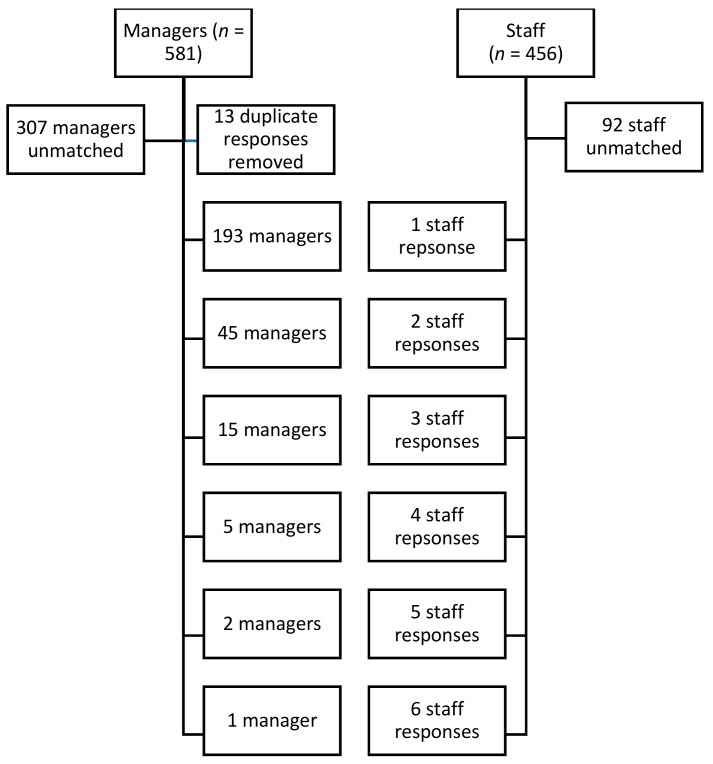
Breakdown of matched manager and staff responses by facility.

Participant demographics (*n* = 1037) are shown in Table 1. Our sample was 97% female (*n* = 942), 66% and 74% of staff and managers had an early childhood educator credential, respectively, 29% of staff had worked in childcare between 1 and 5 years, and just over 65% were between 40 and 59 years old (*n* = 632) based on 456 staff and 581 manager responses from 625 facilities. With an estimated potential sample size of 1514 childcare centers in British Columbia, our overall response rate was 42%. Managers were matched with between 1 and 6 staff from their facility (Figure 1), and in cases where more than one manager response from a facility was received, cases were dropped as described in the Section 2 (*n* = 261 were matched).

We set out to establish prevalence estimates for PA, FMS, and sedentary time policies, practices, and environment and explore whether they were associated with the data provider (manager or staff). Manager-reported prevalence of having PA policies ranged from 40.1% for time children spend outdoors each day to 15.2% for breaking up prolonged sitting (Table 2). Prevalence was significantly associated with whether practices and environment were reported by staff and managers for screen-time, breaking up sitting, and providing FMS activities, and the pattern was consistent for the full and matched samples (see Table 2). The level of agreement and intraclass correlations between matched manager and staff responses are provided in Table 3. Agreement ranged from 55% for providing less than 30 min of screen time up to 74% for providing 60 min of outdoor play. Following a similar pattern, the ICC values for PA practice variables (dichotomous and 5-point scale) were low and ranged from 0.43 and 0.53 for providing 120 min of active play to 0.26 and 0.17 for breaking up prolonged sitting, respectively.

The pattern of associations between policies and PA environment and PA, FMS, and sedentary behavior practices was somewhat similar when practices and descriptors of policies and PA environments were collected from managers or staff and when data from the subsample of managers who had a matched staff person were sampled.

The results from the full sample of managers, subsample of managers, and staff can be found in Table 4, Table 5 and Table 6 respectively and show that engaging in at least 120 min of active play and 60 min of outdoor PA daily was more likely in facilities with enough indoor space for large group running games for all data providers (full and subsample of managers and staff). Having a free-play policy was a significant predictor for the full sample of managers only (Table 4), and when the matched subsample was used, the relationship between the policies and practices disappeared, as well as between FMS activities and indoor space. The staff data (Table 6) only showed a relationship between outdoor space and FMS activities.

Achieving 60 min of outdoor play daily was more likely in centers with policies for the full sample of managers (OR 2.04; 95% CI 1.22–3.52; *p* < 0.01) and in centers with enough outdoor space for large group running games for the full sample of managers (OR 2.74; 95% CI 1.17–6.31; *p* < 0.05), the subsample of managers (OR 6.44; 95% CI 1.93–23.09; *p* < 0.01), and staff (OR 15.3; 05% CI 1.06–221.0; *p* < 0.05 respectively). Daily screen time of less than 30 min was more likely for the full sample of managers in facilities with screen-time policies (OR 1.84; 95% CI 1.21–2.84; *p* < 0.01).

## 4. Discussion

With over half of Canadian children under five years of age utilizing childcare [18] where PA opportunities and FMS promotion is insufficient [19,20,21], it is imperative to understand the impact of state- and facility-level policies on real-world practices. However, concerns about adopting pragmatic policy measurement strategies coupled with “real-world” recruitment challenges related to multi-level data collection and high staff turnover led our team to analyze the variability in childcare facility manager- and staff-reported policies, environments, and practices and the relationship among these factors. Our study is one of few [30,31] that have examined whether prevalence estimates, as they related to policies, descriptions of the environment, and PA, FMS, and sedentary behavior practices, were associated with survey respondent (manager or staff) and whether factors associated with practices differed depending on the source of information.

Our results showed that prevalence estimates were similar between managers and staff with respect to reporting PA practices that are typically scheduled (minutes of active play and time spent playing outdoors) and the PA environment (which is more permanent in nature). This pattern was also similar for the ICCs, as ICC values for PA practices that are typically scheduled were higher, although overall the ICC values indicated a weak agreement between managers and staff. Differences in the prevalence estimates were mainly observed with the reporting of practices that relied more on staff-by-staff implementation, such as those related to sedentary behaviors (screen time and breaking up sitting) and the provision of FMS activities. In these cases, prevalence estimates of meeting the AP standard were higher when data was collected from managers.

Based on Wolfenden et al. [31] and Erinosho et al. [30], we would have expected all of the prevalence estimates to be higher for the manager data; however, our study found that the prevalence estimates were similar for some of the practices and for the description of the environment. Specifically, prevalence estimates between managers and staff were similar for the PA practices (minutes of active play and time spent playing outdoors) and description of the PA environment. Therefore, our findings partially agree with the assertions of Wolfenden et al. [31] and Erinosho et al. [30] as, for some practices, the prevalence of meeting the AP standard was significantly higher when collected from the managers. In addition to factors that were previously noted, the prevalence estimates from the manager data may be higher because (a) managers may feel pressure to report positively to AP standard of practice questions, as they are expected to meet the standards; (b) managers of larger facilities may not be aware of all staff implementation practices across multiple groups of children; (c) implementation is often less than ideal [36]; and (d) as staff turnover is high in childcare facilities, newer staff may not be as familiar with how policies are implemented in practice [36], and there may be variation in staff confidence and competence in providing opportunities to engage in PA and develop FMS [37,38,39]. Similar to assertions by Erinosho et al. [30], the prevalence estimates between managers and staff appeared more consistent when the policies were more specific as well as easier to implement and observe, which may explain why percent agreement and the intraclass correlations for sedentary behaviors (screen time and interruption of sitting) and the provision of FMS activities were less than optimal.

Inconsistencies with respect to these activities may have resulted as some of these activities are irregular (e.g., screen time may not occur every day) or are unlikely to be scheduled into the daily activities (e.g., interruption of sitting will only occur when staff view it as needed versus outdoor time, which may have a planned start and end time). The disagreement related to provision of FMS activities may result from lack of recognition of what counts as an FMS activity. These activities are not necessarily highly structured in nature and can simply involve provision of equipment or engagement in an activity that affords performance of a playful movement foundational to the development of a movement skill, for instance. Alternatively, some staff or managers may have interpreted FMS activities as the provision of scheduled, structured activity delivery, directly designed to encourage a specific movement skill. This is an issue that needs to be explored further, possibly through interviewing childcare managers and staff.

When we examined whether the patterns of associations among policies, environmental factors, and PA, FMS, and sedentary practices were consistent across manager and staff data, the results were somewhat consistent between the subsample of managers and staff, with one notable exception—the association with FMS practices. This may be the result of the issues described previously. Although more associations were noted in the full sample of managers, many associations disappeared in the subsample of managers who had a staff match, rendering the results of the manager and staff data more comparable. Overall, this study found evidence that the prevalence estimates and associations with policy and environment from manager responses were similar to staff responses for PA-related practices, thus supporting the use of either staff or manager responses in the surveillance context. 

Although we set out to explore the feasibility of manager-only data for monitoring a state-level policy, our results also highlighted important relationships between policy and practice. The full manager sample showed that the existence of policies in line with the AP standard were associated with better practices for four of the practices examined, but no association was observed for the FMS skills only. While these effects disappear in the matched subsample of managers and staff, we suspect that the decrease in sample size likely explains these differences. Interestingly, our study found more consistency between having a policy and implementing practices than previous studies [30,40], although the literature is inconsistent. For example, Bower et al. [40] found a weak relationship between policy and more PA time, but Erinosho et al. [30] found that the presence of a policy was related to less PA time (as measured through direct observations) for children in childcare. Similarly, a recent systematic review by Vanderloo et al. [41] determined that the evidence supporting policy as a potential correlate of screen-viewing among preschoolers in childcare was inconclusive. This review highlighted wide variation in the measurement and operationalization of screen-time policy, as well as access to and use of screens across studies in childcare [41]. Our findings may in part be due to measurement of self-reported practices versus direct assessment of child and staff behaviors. As well, our measurements were taken in a childcare monitoring setting where both practices and policies are monitored by the overseeing organization (Director of Licensing) every 18 months (more often after a contravention). This dual oversight may result in more consistency in comparison to research settings where there is no state-level accountability or monitoring of practices and policies or where the monitoring of these two pieces are done by different organizations.

Our study found that practices were also related to environmental infrastructure, with large indoor and/or outdoor spaces supporting PA, FMS, and positive sedentary behaviors. This was consistent in the matched sample, except for the association between outdoor spaces and FMS activities, which was only observed in staff data. This is in agreement with previous research that showed associations between PA and larger indoor spaces, larger outdoor play areas, and equipment availability in the outdoor area [40,42]. Interestingly, the only notable dissimilarity in associations was observed for breaking up prolonged sitting (difference in prevalence of ~21%). Overall, both our manager and staff results support the notion that large play spaces are supportive of movement behaviors in a childcare setting.

As with any studies, the results of this study should be interpreted in light of its limitations. First, the use of self-report to assess policies, practice, and description of the environment is known to be associated with measurement error. Second, while a census sampling approach was used to recruit participants (all site managers were invited to fill out the survey), participants had to volunteer to participate, and as such, the prevalence estimates may be positively biased. Responses from individual providers may also not be representative of the entire center, as provider practices, expertise and confidence, and perceived role and professional identity (seeing it as important) have been shown to vary in previous research with teachers [43]. Our findings should be viewed in light of the differences in experience between childcare managers and staff, as it has been previously reported that 39.3% of the childcare managers in this study had worked in childcare for more than 20 years [44], while 35.9% of the staff had worked in childcare less than 5 years. Recent entrants to the field are likely to be exposed to the concepts associated with physical literacy during their training, but less experience working in childcare could cause them to have more difficulty successfully implementing the practices. Lastly, our study focused on policy-relevant intermediate practices that theoretically directly link to child behaviors, but no child-level PA, FMS, or other physical literacy components were measured. There is a need to replicate these findings in a study where child-level data are collected, as direct measurement of these variables may lead to different results. The strengths of the study include basing our questions on the EPAO-SR tool, which has been shown to be reliable and valid [28] and our response rate, which was commensurate with other real-world surveys [45].

## 5. Conclusions

Reaching childcare staff to participate and high staff turnover present significant challenges for longitudinal survey research and monitoring of policy impact. Based on our data and the data collected by others, the differences in responses between managers and staff may continue to be a challenge for AP policy and practice research. The prevalence estimates of policy and practice reported by managers alone appear to be a modest overestimation in comparison to staff-reported policy and practices [32,34]; however, using data from either appears to be a viable alternative for pragmatic monitoring of policy impact (surveillance).

Our data showed that childcare facility-level policy and physical environment matters. Written policy and indoor and outdoor space were associated with meeting recommended daily PA and sedentary practices. However, policy change may be essential for driving practice change in settings without supportive physical environments. Coupling this with professional development for managers and staff may improve policy implementation and their relationship with implementation. Further exploration of FMS policies and their implementation is needed to understand what they entail and to examine staff awareness and understanding of facilitating FMS as a distinct practice. Finally, exploration of educator training, including examination of knowledge and self-efficacy for teaching FMS and providing PA opportunities, should be conducted.

## Figures and Tables

**Table 1:** Demographic characteristics of childcare facility respondents.

	% and Sample Size
**Age in years (*n* = 970)**	
20–39	13.7%; (*n* = 133)
40–59	65.2%; (*n* = 632)
60+	8.7%; (*n* = 84)
**Gender (*n* = 971)**	
Female	97.0%; (*n* = 942)
Male	2.2%; (*n* = 21)
Prefer not to disclose	0.0%; (*n* = 8)
Staff	44.0%; (*n* = 456)
Manager	56.0%; (*n* = 581)
**ECE Credential (*n* = 968)**	
Manager	73.8%; (*n* = 429)
Staff	66.0%; (*n* = 301)
**Staff years worked in childcare (*n* = 416)**	
<1	7.0%; (*n* = 32)
1–5	28.9%; (*n* = 132)
6–9	15.1%; (*n* = 69)
10–19	24.3%; (*n* = 111)
20+	15.8%; (*n* = 72)

Note: ECE = early childhood educator.

**Table 2:** Prevalence of reported physical activity policies, practices, and environment by childcare facility managers and staff.

Active Play Standards of Practice	Managers—Full Sample (*N* = 581)	Managers—Sub Sample (*N* = 261)	Staff (*N* = 456)	Chi-Square Test—Full Sample		Chi-Square Test—Sub Sample		
				χ^2^	*p* Value	χ^2^	*p* Value	
120 min active play	67.70%	70.60%	70.40%	0.72	0.4	0.01	0.99	
≤30 min on screens	46.60%	47.90%	37.20%	7.64	0.01	6.25	0.01	
FMS activities	76.70%	79.30%	61.40%	23.62	0	19.1	0	
Breaking up sitting	75.80%	77.00%	55.60%	34.39	0	22.9	0	
60 min of outdoor play	76.40%	79.60%	77.80%	0.35	0.85	0.51	0.48	
**Physical Activity Environment**								
Indoor space for large group running games	28.90%	29.40%	29.60%	0.18	0.67	0.04	0.84	
Outdoor space for large group running games	92.70%	93.30%	90.00%	1.17	0.28	1.13	0.29	
**Policies related to Active Play**								
The amount of unfacilitated play/free play	30.60%	28.30%	--					
The amount of time children can play with screens	26.30%	27.80%	--					
The amount of activities targeting FMS	23.10%	19.80%	--					
Breaking up prolonged sitting time with activity	15.20%	12.60%	--					
The amount of time children spend outdoors each day	40.10%	38.90%	--					

Notes: FMS = fundamental movement skills.

**Table 3:** Agreement and intraclass correlation (ICC) between managers and staff for dichotomous data.

	Agreement	ICC Dichotomous	ICC 5-Point Scale
120 min active play	65%	0.40	0.53
≤30 min screen time	55%	0.27	0.24
FMS activities	57%	0.21	0.31
Breaking up sitting	58%	0.26	0.17
60 min of outdoor play	74%	0.43	0.36

**Table 4:** Policy and environmental correlates of Active Play standards for the full sample of childcare facility managers.

	120 Min of Active Play		30 Min or Lesson Screens		Activities that Develop FMS		Breaking up Prolonged Sitting		60 Min of Outdoor Active Play	
	Odds Ratio (95% CI)	*p*	Odds Ratio (95% CI)	*p*	Odds Ratio (95% CI)	*p*	Odds Ratio (95% CI)	*p*	Odds Ratio (95% CI)	*p*
Policy	2.19 (1.39–3.52)	0.001	1.84 (1.21–2.84)	0.005	--	--	2.29 (1.14–5.15)	0.029	2.04 (1.22–3.52)	0.008
Indoor space	1.99 (1.24–3.27)	0.005	--	--	2.09 (1.23–3.69)	0.009	--	--	--	--
Outdoor space	--	--	--	--	--	--	2.33 (1.08–4.91)	0.014	2.74 (1.17–6.31)	0.018

Notes: -- = variables dropped to create most parsimonious models.

**Table 5:** Policy and environmental correlates of Active Play standards for the sample of childcare facility managers matched with staff responses.

	120 Min of Active Play		30 Min or Lesson Screens		Activities that Develop FMS		Breaking up Prolonged Sitting		60 Min of Outdoor Active Play	
	Odds Ratio (95% CI)	*p*	Odds Ratio (95% CI)	*p*	Odds Ratio (95% CI)	*p*	Odds Ratio (95% CI)	*p*	Odds Ratio (95% CI)	*p*
Policy	--	--	--	--	--	--	--	--	--	--
Indoor space	2.45 (1.18–5.54)	0.022	--	--	--	--	--	--	--	--
Outdoor space			--	--			--	--	6.44 (1.93–23.09)	0.003

Notes: -- = variables dropped to create most parsimonious models.

**Table 6:** Policy and environmental correlates of Active Play standards for childcare facility staff.

	120 Min of Active Play		30 Min or Lesson Screens		Activities that Develop FMS		Breaking up Prolonged Sitting		60 Min of Outdoor Active Play	
	Odds Ratio (95% CI)	*p*	Odds Ratio (95% CI)	*p*	Odds Ratio (95% CI)	*p*	Odds Ratio (95% CI)	*p*	Odds Ratio (95% CI)	*p*
Policy	--	--	--	--	--	--	--	--	--	--
Indoor space	7.68 (1.1–53.7)	0.040	--	--	--	--	--	--	--	--
Outdoor space			--	--	2.41 (1.08–5.38)	0.032	--	--	15.3 (1.06–221.0)	0.045

Notes: -- = variables dropped to create most parsimonious models.

## Appendix A

**Table A1:** Eight standards of practice for active play in BC childcare centers.

Director of Licensing Standard of Practice for Active Play (AP standard)
1. Ensure a minimum of 60 min per day of outdoor active play (indoor active play is acceptable when weather is poor or outdoor physical space is limited). Active play may be accumulated through 15-min portions of time throughout the day or continuously.
2. Licensees and employees must be aware of and incorporate fundamental movement skills and injury prevention into all active play activities.
3. A licensed preschool care program must ensure the minimum outdoor active play corresponds with the length of time the preschool program is offered: 1–2 h, 20 min; 2–3 h, 30 min; 3–4 h, 40 min.
4. All licensed childcare programs must limit screen time (TV, computer, electronic games) to 30 min or less a day.
5. Licensees and employees must limit prolonged sitting activities (in a stroller, highchair, board games, crafts) and schedule frequent short bursts of activity for one to two minutes.
6. Employees must demonstrate appropriate modeling of active play activities and screen time.
7. The licensee must develop and implement an active play policy to engage children in daily active play, consisting of unfacilitated play and facilitated games and activities. This policy should also be shared with parents/families.
8. The licensee must develop and implement a screen use policy to guide employees in the use of screen-time activities. This policy should also be shared with parents/families.
